# Bovine Immunodeficiency Virus as a potentiating cofactor for the experimental Bovine Leukemia Virus infection in sheep

**DOI:** 10.1186/1742-4690-8-S1-A5

**Published:** 2011-06-06

**Authors:** Marzena Rola, Bozenna Kozaczynska, Aneta Pluta, Jacek Kuzmak

**Affiliations:** 1 Department of Biochemistry, National Veterinary Research Institute, Pulawy, 24-100, Poland

## 

Coinfection with bovine retroviruses Bovine Leukemia Virus (BLV) and Bovine Immunodeficiency Virus (BIV) is currently noted in the naturally infected cattle. The effect of BIV as a cofactor for the BLV infectivity is still unknown. In our study we examined the impact of BIV on the BLV in experimentally infected sheep. Four sheep were inoculated with Foetal Bovine Lung cells (FBL) infected with the R29 isolate of BIV and the other four, mock-infected, received uninfected FBL cells. Two weeks later all animals were inoculated intravenously with 5x10^5^ of PBMC of cow persistently infected with BLV. Blood samples were collected every 4 week up to 40th week after inoculation (a.i.). From 4th up to 40th week a.i. antibody titer to BLV in dually infected sheep were almost 1.0 log10 higher than those in BLV group. The BLV infectivity, quantified by BLV-induced syncytia, revealed that the numbers of syncytia in BLV/BIV inoculated animals were higher then those in sheep inoculated with BLV alone. BLV proviral load, determined by a qPCR, increased in the doubly infected animals from 16th to 28th and between 36th and 40th week a.i. Finally semiquantitative RT-PCR disclosed significant increment of BLV tax mRNA at 36th and 40th weeks a.i. in BIV/BLV inoculated sheep. In conclusion these findings confirmed that BIV infection enhance expression of BLV infection in experimentally inoculated sheep.

